# Activity of Omadacycline in Rat Methicillin-Resistant Staphylococcus aureus Osteomyelitis

**DOI:** 10.1128/AAC.01703-21

**Published:** 2022-01-18

**Authors:** Melissa J. Karau, Suzannah M. Schmidt-Malan, Scott A. Cunningham, Jayawant N. Mandrekar, Bobbi S. Pritt, Tiffany R. Keepers, Alisa W. Serio, Surya Chitra, Robin Patel

**Affiliations:** a Division of Clinical Microbiology, Department of Laboratory Medicine and Pathology, Mayo Clinic, Rochester, Minnesota, USA; b Division of Biomedical Statistics and Informatics, Department of Health Sciences Research, Mayo Clinic, Rochester, Minnesota, USA; c Paratek Pharmaceuticals, Inc., King of Prussia, Pennsylvania, USA; d Division of Infectious Diseases, Department of Medicine, Mayo Clinic, Rochester, Minnesota, USA

**Keywords:** omadacycline, vancomycin, rifampin, osteomyelitis, methicillin-resistant *Staphylococcus aureus*

## Abstract

Omadacycline, vancomycin, and rifampin, as well as rifampin combination therapies, were evaluated in an experimental rat model of methicillin-resistant Staphylococcus aureus (MRSA) osteomyelitis. All treatment groups had less MRSA recovered than saline-treated animals. The emergence of rifampin resistance was observed in 3 of 16 animals with rifampin monotherapy and none with rifampin combination therapy. After treatment, the median tibial bacterial loads were 6.04, 0.1, 4.81, and 5.24 log_10_ CFU/g for saline-, rifampin-, vancomycin-, and omadacycline-treated animals, respectively. Omadacycline or vancomycin administered with rifampin yielded no detectable MRSA. Omadacycline administered with rifampin deserves evaluation in humans as a potential treatment for osteomyelitis.

## INTRODUCTION

Staphylococcus aureus, the most common cause of osteomyelitis, which often requires surgical intervention and long-term antimicrobial therapy (1–3), has the ability to evade the immune system and antibiotics by forming protective biofilms (4, 5), surviving intracellularly in several cell types (6–8) and producing a plethora of virulence factors (3). Compounding this situation has been an increase in drug resistance in this species, with many available antimicrobials having poor activity against staphylococcal biofilms (5, 9, 10) and/or lacking the ability to penetrate bone tissue/cells (6).

Omadacycline is an aminomethylcycline protein synthesis inhibitor designed to overcome efflux and ribosomal protection mechanisms associated with tetracycline resistance (11, 12). It has a broad spectrum of activity against aerobic bacteria, including methicillin-resistant S. aureus (MRSA), vancomycin-resistant enterococci, extended-spectrum-β-lactamase-producing *Enterobacterales* strains, multidrug-resistant pneumococci, and *Legionella* species, as well as anaerobes and *Mollicutes* strains (7, 13–16). Oral and intravenous formulations of omadacycline have been approved by the U.S. Food and Drug Administration (FDA) for treatment of acute bacterial skin and skin structure infections (ABSSSIs) and community-acquired bacterial pneumonia caused by certain bacteria, including S. aureus. Omadacycline has shown *in vivo* activity in murine intraperitoneal and murine thigh MRSA infection models and has demonstrated antibiofilm activity against Escherichia coli and Staphylococcus species and activity against S. aureus-infected monocytes *in vitro* (7, 17–21). Moreover, a study by Lin et al. showed that rat bone mineral had the highest tissue-to-blood concentrations of the tissues examined, after intravenous administration of 5 mg/kg omadacycline (22). This raised the possibility that omadacycline might provide a treatment option for bone infections; therefore, the activity of omadacycline in an experimental MRSA osteomyelitis model was investigated.

## RESULTS

MICs and minimum bactericidal concentrations (MBCs) for the study strain MRSA IDRL-6169 were 0.5 and >32 μg/ml for omadacycline, 1 and >32 μg/ml for vancomycin, and 0.004 and 1 μg/ml for rifampin, respectively (Table 1). Minimum biofilm inhibitory concentrations (MBICs) and minimum biofilm bactericidal concentrations (MBBCs) were 0.5 and >64 μg/ml for omadacycline, 2 and >128 μg/ml for vancomycin, and 0.008 and 4 μg/ml for rifampin, respectively (Table 1). The *in vitro* activities of omadacycline, vancomycin, and rifampin were similar for MRSA IDRL-4293 and S. aureus ATCC 29213, except that MRSA IDRL-4293 was rifampin resistant (Table 1).

**TABLE 1:** Antimicrobial susceptibility of MRSA IDRL-6169, MRSA IDRL-4293, and S. aureus ATCC 29213

Strain and drug	MIC (μg/ml)	MBC (μg/ml)	MBIC (μg/ml)	MBBC (μg/ml)
*S. aureus* IDRL-6169				
Vancomycin	1	>32	2	>128
Rifampin	0.004	1	0.008	4
Omadacycline	0.5	>32	0.5	>64
*S. aureus* IDRL-4293				
Vancomycin	1	4	8	>128
Rifampin	>16	>16	>16	>16
Omadacycline	0.5	64	2	64
*S. aureus* ATCC 29213				
Vancomycin	1	4	2	>128
Rifampin	0.008	0.25	0.008	>4
Omadacycline	1	64	2	64

**FIG 1:**
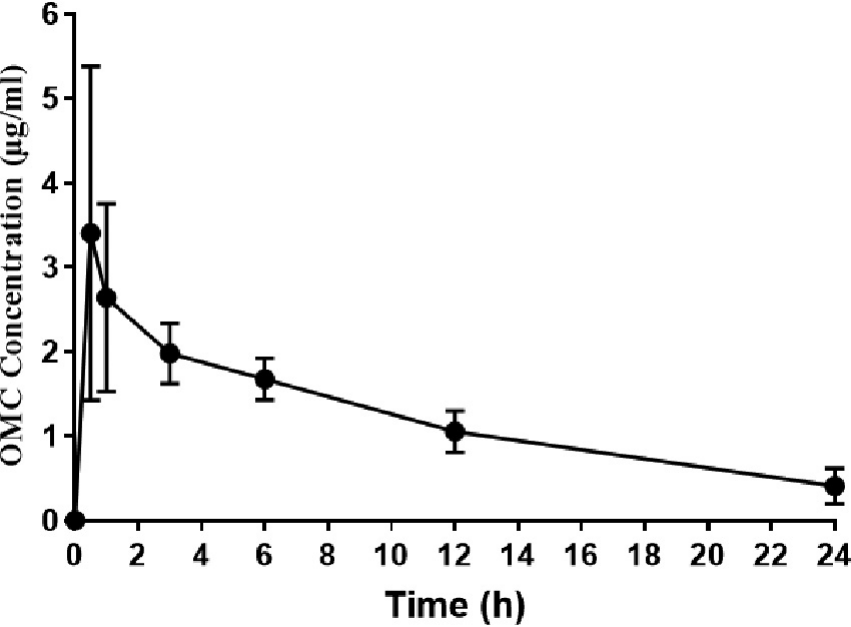
Omadacycline (OMC) mean plasma concentrations after a single 20-mg/kg intraperitoneal dose in 4 healthy rats. The *C*_max_ was 3.4 μg/ml at 0.5 h, and the AUC_0–24_ was 29.4 μg · h/ml.

Mean omadacycline plasma concentrations versus time after a single 20 mg/kg dose of omadacycline in healthy, uninfected rats are shown in Fig. 1. Based on this concentration versus time profile, the maximum concentration (*C*_max_) was calculated to be 3.4 μg/ml at 0.5 h, and the area under the concentration-time curve from 0 to 24 h (AUC_0–24_) was 29.4 μg · h/ml. Mean plasma concentrations of omadacycline in infected rats were evaluated 2 h after administration of drug on treatment days 1, 3, and 10. Mean concentrations of omadacycline on days 1, 3, and 10 were 3.9 ± 0.84, 0.89 ± 0.78, and 0.74 ± 0.11 μg/ml, respectively, with an average of 1.8 ± 1.6 μg/ml across all days. Mean concentrations of omadacycline, when administered with rifampin, on days 1, 3, and 10 were 4.7 ± 0.62, 1.53 ± 0.89, and 2.6 ± 1.6 μg/ml, respectively, with an average of 2.9 ± 1.7 μg/ml across all days. Compared to the plasma concentration profile of healthy animals (mean omadacycline concentrations of 2.6 ± 0.92 μg/ml at 1 h and 2.0 ± 0.3 μg/ml at 3 h), these values suggest no effect of infection on omadacycline plasma concentrations in this model; while omadacycline concentrations trended slightly higher when omadacycline was administered with rifampin, conclusions cannot be drawn from the limited data set.

Omadacycline dosing in rats resulted in slightly higher pharmacokinetic values than reported for humans. For example, the steady-state AUC in healthy human adults ranged from 11.2 to 13.7 μg · h/ml for FDA-approved doses and routes of administrations (100 mg intravenously, 300 mg orally, and 450 mg orally) (23). In rats, the rifampin AUC_0–24_ was previously determined to be 332 μg · h/ml, with a peak concentration of 28 μg/ml (24) (normal human *C*_max_, 8 to 24 μg/ml [25]). A vancomycin AUC/MIC ratio between 400 and 600 is recommended for humans (26); with a MIC of 1 μg/ml, the vancomycin AUC/MIC was 368, slightly lower than this goal range. However, higher doses in rats have been shown to cause toxicity (27).

**FIG 2:**
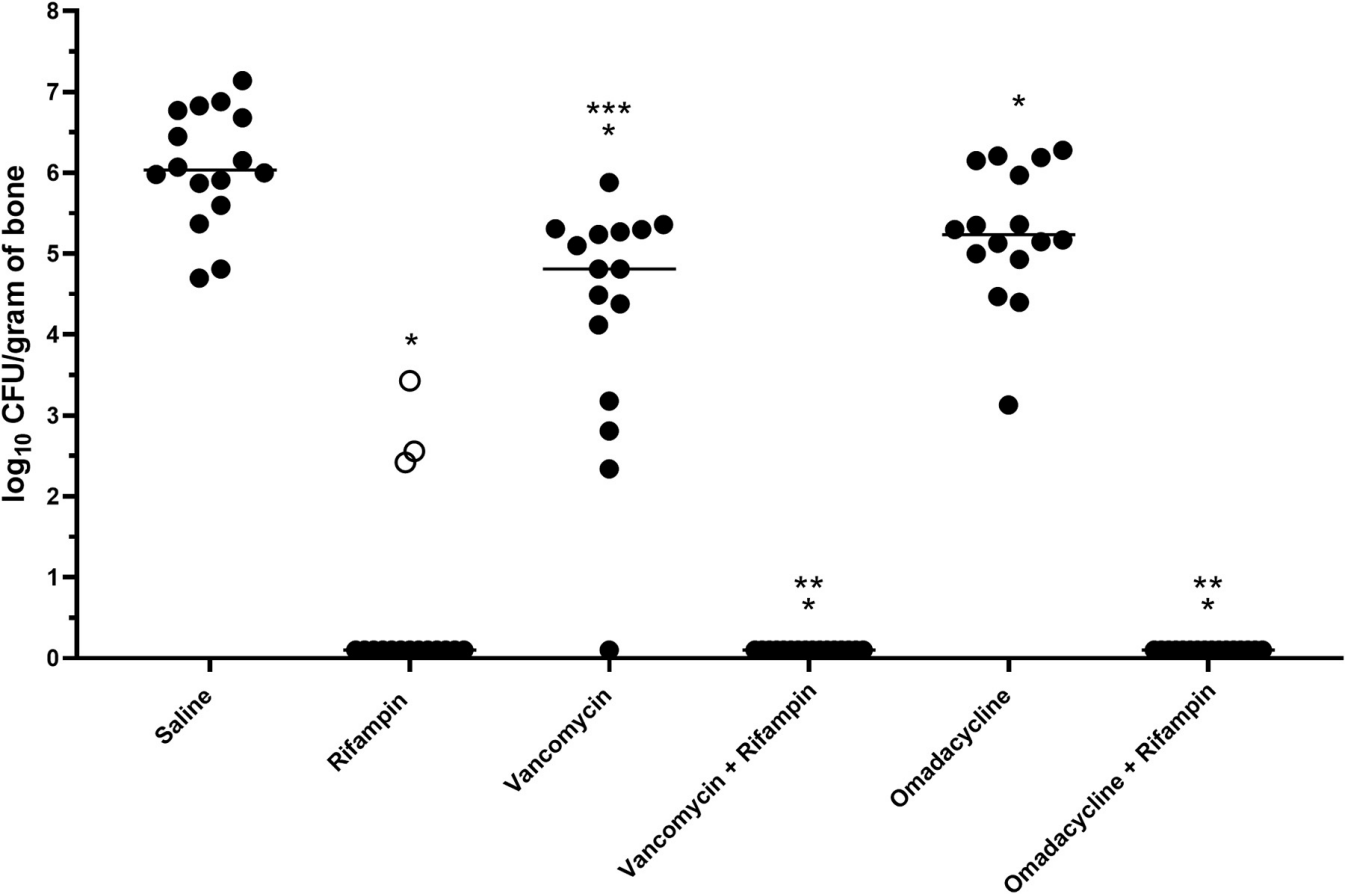
Amounts of MRSA recovered from the left tibiae after 21 days of treatment. Each dot represents the value from 1 animal, and the horizontal lines represent median values. The open dots indicate recovery of rifampin-resistant MRSA. Significant reductions are indicated as follows. *, All groups versus saline (favors treatment, *P* ≤ 0.0122). **, Rifampin combination therapy versus vancomycin or omadacycline alone (favors combination therapy, *P* < 0.0001). ***, Vancomycin versus omadacycline (favors vancomycin, *P* < 0.0348).

Results of bone cultures for each animal are shown in Fig. 2. The median amounts of MRSA were 6.04 log_10_ CFU/g (range, 4.7 to 7.14 log_10_ CFU/g), 0.10 log_10_ CFU/g (range, 0.1 to 3.43 log_10_ CFU/g), 4.81 log_10_ CFU/g (range, 0.1 to 5.88 log_10_ CFU/g), and 5.24 log_10_ CFU/g (range, 3.13 to 6.28 log_10_ CFU/g) for the saline-, rifampin-, vancomycin-, and omadacycline-treated groups, respectively (Fig. 2). No MRSA was recovered in either of the rifampin combination groups. All groups had significantly less MRSA recovered than saline-treated animals (*P* ≤ 0.0122). The amounts of MRSA recovered with vancomycin monotherapy were lower than those with omadacycline monotherapy (*P* = 0.0348). Rifampin monotherapy was not significantly different than combination therapies; however, the emergence of rifampin resistance was detected in 3 animals in the rifampin monotherapy group (MICs of >16 μg/ml).

**FIG 3:**
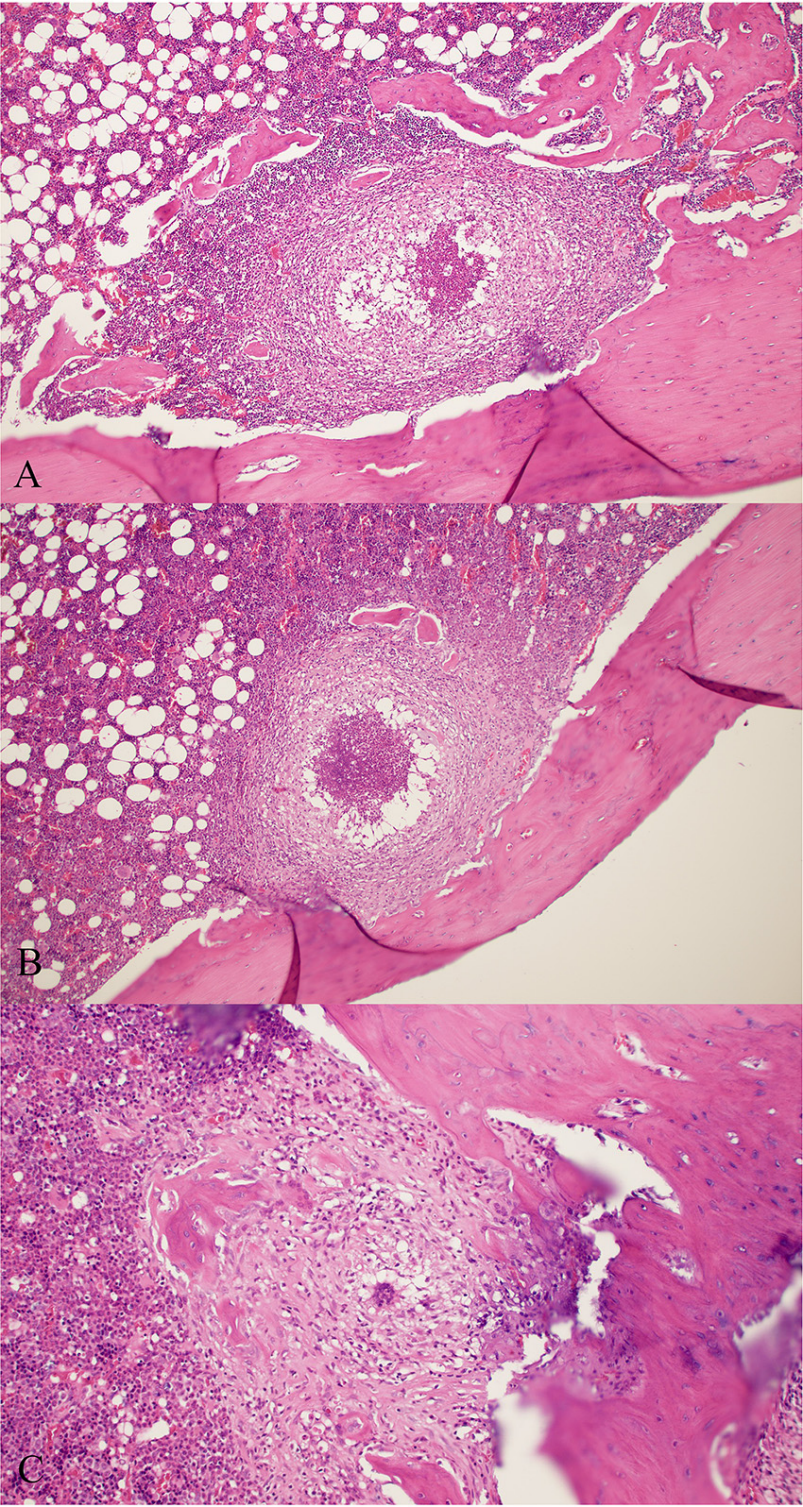
(A and B) Granuloma with central suppuration observed in saline-treated (A) and vancomycin-treated (B) animals. (C) Multinucleated giant cell observed in an animal treated with omadacycline plus rifampin. Magnification, ×10.

The physical appearance of animals in the different treatment groups was not different. Yellowing of the bone (the color of the omadacycline) was noted in omadacycline-treated animals. Histopathologic examination showed that saline- and vancomycin-treated animals each had a single granuloma with central suppuration (Fig. 3A and B). A multinucleated giant cell was seen in an animal treated with omadacycline plus rifampin (Fig. 3C). Fibrosis (injection artifact) was noted in the omadacycline-, rifampin-, and rifampin combination-treated animals, with no granulomas, abscesses, or bacteria seen (data not shown).

## DISCUSSION

Staphylococcal osteomyelitis is a devastating disease associated with high morbidity rates and cost. In pediatric *S. aureus* osteomyelitis, methicillin-resistant cases have been reported to have worse outcomes than methicillin-susceptible cases, including longer hospital stays, more surgeries, recurrent infections, and higher morbidity rates (28). Fast-acting, effective antimicrobials are lacking. Ideally, antimicrobial agents should have high antibiofilm activity and bone penetration to effectively reach staphylococci protected in biofilm matrixes, bone tissue, and cells.

Omadacycline has demonstrated intracellular activity against S. aureus-infected human monocytes, in which a ≥2-log_10_ CFU/ml reduction was observed at 24 h at 2× the MIC (7). In the same study, omadacycline MICs against S. aureus, including MRSA, ranged from 0.016 to 1 μg/ml, with a MIC_90_ of 0.25 μg/ml, and extracellular bactericidal activity was observed, with ≥3-log_10_ CFU/ml reductions at both 1× and 6× the MIC after 24 h. Omadacycline demonstrated activity against E. coli biofilms *in vitro*, reducing the total bioburden at concentrations close to the MIC, and did not induce E. coli biofilm formation at sub-MIC concentrations (20). Additionally, a recent publication demonstrated that omadacycline alone and in combination with rifampin prevented S. aureus and Staphylococcus epidermidis biofilm formation *in vitro* (21). Omadacycline had low biofilm MICs, ranging from 0.5 to 1 μg/ml for S. aureus and from 0.25 to >16 μg/ml for S. epidermidis, with synergistic activity being observed in combination with rifampin in 75% of the strains in biofilm time-kill assays (21). Furthermore, omadacycline prevented the emergence of rifampin resistance observed in one of the S. epidermidis strains tested in a biofilm reactor model (21). Omadacycline was tested against the study strain (MRSA IDRL-6169) and two other strains, and the MBIC of the study strain was found to be equivalent to the MIC (0.5 μg/ml); however, no bactericidal activity was observed. Vancomycin, an antibiotic that is recommended for the treatment of orthopedic MRSA infections, also did not show bactericidal activity *in vitro*.

In a pharmacokinetic study by Lin et al., the highest tissue-to-blood concentrations were found in bone mineral following administration of a single 5-mg/kg dose of ^14^C-labeled omadacycline (22). The tetracycline drug class has high affinity for cations such as magnesium and calcium and can form insoluble complexes (29). It is possible that omadacycline is binding to calcium in bone and that this may contribute to the decreased efficacy observed when omadacycline was administered as monotherapy, compared to vancomycin monotherapy, in this model. These studies combined suggest that, due to extracellular and intracellular activity and bone penetration, omadacycline deserves further evaluation as an option for treating orthopedic infections, such as osteomyelitis in humans.

Both vancomycin and omadacycline alone were more active than saline alone; however, >4 log_10_ CFU/g (median) of MRSA was still recovered after 21 days of treatment. Currently available antibiotics are often less than perfect in the treatment of orthopedic infections because of the complex nature of the bone. Addition of rifampin is recommended in some scenarios due to its activity against staphylococcal biofilms (30, 31) and intracellular staphylococci (31) and its ability to penetrate bone tissue (32). We have shown in multiple studies that rifampin is active in orthopedic MRSA rat models; however, we have observed several instances of selection of rifampin resistance with rifampin monotherapy (24, 27, 33), including 3 animals in the current study. The rate of reported emergence of rifampin resistance varies from study to study, both in our work and in the literature, varying with rifampin dose and model type. Clinically, it is not recommended that rifampin be used alone, because of the potential emergence of resistance and consequent treatment failure (1, 34). When it is used in combination with another antistaphylococcal antibiotic, treatment is more effective in eradicating the infection. When omadacycline is administered with rifampin, it offers the advantage of leveraging rifampin’s bactericidal activity and the ability of both agents to penetrate bone and to reach intracellular bacteria, while limiting the chance of selection of resistance. Additionally, omadacycline is orally bioavailable and has an FDA-approved oral formulation. In this study, when omadacycline was administered with rifampin, MRSA was eradicated in all animals and no resistance was observed.

There are several limitations to this study. First, this model was a model of chronic osteomyelitis, which may be harder to treat than acute infection. Second, because no surgical intervention was included prior to the initiation of antimicrobial treatment, the model was more stringent than the usual clinical strategy; osteomyelitis typically requires irrigation and debridement of infected, necrotic bone tissue in addition to long-term antimicrobial therapy (1, 3). Third, only one strain of MRSA was tested. Fourth, while omadacycline is known to penetrate bone and to interact with bone materials such as calcium, it was beyond the scope of the current study to determine whether omadacycline remains active within bone.

In conclusion, omadacycline showed activity in a rat chronic MRSA osteomyelitis model when administered alone, with higher activity when it was administered with rifampin, abrogating the emergence of rifampin resistance observed with rifampin monotherapy. Omadacycline and rifampin combination therapy deserves further evaluation as a potential treatment option for human MRSA osteomyelitis.

## MATERIALS AND METHODS

The strain studied *in vivo* is a clinical MRSA isolate (IDRL-6169) that was isolated from a periprosthetic hip infection at Mayo Clinic (Rochester, MN) and has been shown to produce infection in previous osteomyelitis studies (24, 27, 33, 35–39). Susceptibility testing was performed with omadacycline (Paratek Pharmaceuticals, Inc., Boston, MA), vancomycin (Sigma-Aldrich, St. Louis, MO), and rifampin (Sigma-Aldrich), as follows. MICs and MBCs were determined following CLSI guidelines (34, 40, 41). MBICs and MBBCs were determined using a pegged-lid assay, as described previously (42). In addition to IDRL-6169, MRSA IDRL-4293 and S. aureus ATCC 29213 were tested *in vitro*.

The study was approved by the Mayo Clinic Institutional Animal Care and Use Committee. Omadacycline plasma concentrations were determined in healthy Sprague-Dawley rats (Envigo, Indianapolis, IN) after a single 20 mg/kg intraperitoneal dose of omadacycline. Blood was collected from 4 rats via the tail vein at 0.5, 1, 3, 6, 12, and 24 h after omadacycline administration, and plasma concentrations of omadacycline were analyzed at Q^2^ Solutions (Ithaca, NY) by TurboIonSpray liquid chromatography-tandem mass spectrometry. Mean omadacycline plasma concentrations versus tested time points for uninfected Sprague-Dawley rats were used to estimate the pharmacokinetic parameters (*C*_max_ and AUC_0–24_) of omadacycline in plasma by standard noncompartmental methods using a WinNonlin (Certara, Princeton, NJ)-validated SAS program.

Experimental chronic osteomyelitis was established in 102 Sprague-Dawley rats using a previously described model of rat osteomyelitis (43). Briefly, rats were anesthetized, the left leg was shaved and disinfected, and a 1-cm incision was made over the medial portion of the proximal tibia. A 1.5-mm hole was bored into the tibia, and 10 μl of arachidonic acid (50 μg/ml) and 60 μl of 10^8^ CFU/ml MRSA IDRL-6169 were injected into the medullary cavity. The hole was sealed with dental gypsum, and the site was closed. Four weeks after the establishment of infection, rats were randomly assigned to one of six intraperitoneal treatment arms (17 animals/group), as follows, and treated for 21 days: saline (∼0.7 ml, once daily), rifampin (25 mg/kg, twice daily), vancomycin (75 mg/kg, twice daily), omadacycline (20 mg/kg, once daily), vancomycin plus rifampin, and omadacycline plus rifampin. Plasma was collected 2 h after treatment from 4 animals each in the omadacycline and omadacycline plus rifampin groups at 1, 3, and 10 days, to determine plasma omadacycline levels in infected animals.

Twelve hours after completion of vancomycin and rifampin therapy and 24 h after saline and omadacycline therapy, rats were euthanized, and the left tibiae were aseptically removed. One infected tibia from each group was chosen for histopathologic examination, fixed for 48 h in 10% formalin, cut in half longitudinally, paraffin embedded with the medullary cavity surface on top, sectioned longitudinally, mounted on glass slides, and stained with hematoxylin and eosin. Slides were reviewed by a board-certified pathologist with expertise in microbiology and infectious disease pathology. The remaining tibiae were cryopulverized and weighed for quantitative bacterial culture. In addition, to screen for the emergence of resistance, bone homogenates were plated on Mueller-Hinton agar (MHA) containing 4 μg/ml of rifampin, vancomycin, or omadacycline if the animals had received treatment with the cognate antibiotic. All colonies recovered on selective medium were subjected to identification and MIC testing with the relevant antibiotic.

Results were reported as log_10_ CFU per gram of bone. Descriptive summaries are reported as median and range. Comparisons among the six groups were first performed using the Kruskal-Wallis test. Due to statistically significant differences between the groups, further comparisons between groups were performed in a pairwise manner using the Wilcoxon rank sum test. Nonparametric tests were used because of the small sample size and non-normally distributed data. All tests were two sided, and *P* values of <0.05 were considered statistically significant. Analysis was performed using SAS software version 9.4 (SAS Inc., Cary, NC).
